# IL-1-IL-17 Signaling Axis Contributes to Fibrosis and Inflammation in Two Different Murine Models of Systemic Sclerosis

**DOI:** 10.3389/fimmu.2018.01611

**Published:** 2018-07-10

**Authors:** Min-Jung Park, Su-Jin Moon, Eun-Jung Lee, Kyung-Ah Jung, Eun-Kyung Kim, Da-Som Kim, Jung-Ho Lee, Seung-Ki Kwok, Jun-Ki Min, Sung-Hwan Park, Mi-La Cho

**Affiliations:** ^1^The Rheumatism Research Center, Catholic Research Institute of Medical Science, The Catholic University of Korea, Seoul, South Korea; ^2^Divison of Rheumatology, Department of Internal Medicine, The Catholic University of Korea, Seoul, South Korea; ^3^Department of Plastic and Reconstructive Surgery, College of Medicine, The Catholic University of Korea, Seoul, South Korea

**Keywords:** systemic sclerosis, skin fibrosis, lung fibrosis, interleukin-17, interleukin-1, chronic graft-versus-host disease

## Abstract

**Objective:**

Systemic sclerosis (SSc) is a progressive fibrotic disease that affects the skin and internal organs. Despite evidence implicating increased interleukin-17 (IL-17) activity in SSc, the role of IL-17 in SSc remains uncertain. The purpose of this study was to investigate whether IL-17 plays a pathophysiological role in SSc in two different murine models of SSc.

**Methods:**

Bleomycin (BLM)-induced fibrosis and chronic graft-versus-host disease (cGVHD) models were used. Histological analysis was performed using Masson’s trichrome and immunohistochemical staining. Quantitative reverse transcription-polymerase chain reaction and enzyme-linked immunoassays were used to quantify the messenger RNA and protein levels of inflammatory mediators in dermal fibroblasts.

**Results:**

IL-1 receptor antagonist-deficient (IL-1Ra-KO) mice were more severely affected by BLM injection, as shown by dermal and pulmonary fibrosis, compared with wild-type (WT) mice. Increased tissue fibrosis was reversed by knocking down IL-17. *In vitro* experiments showed that IL-1 and IL-17 exerted synergistic effects on the expression of profibrotic and inflammatory mediators. In the cGVHD model, C57BL/6 mice receiving splenocytes of IL-1Ra-KO BALB/c mice developed more severe cGVHD than did those receiving cells from WT mice. Knockdown of IL-17 in IL-1Ra-KO donor mice significantly attenuated the IL-1-induced acceleration of cGVHD severity.

**Conclusion:**

Targeting IL-1 and its downstream IL-17 activity may be a novel treatment strategy for inhibiting inflammation and tissue fibrosis in SSc.

## Introduction

Systemic sclerosis (SSc) is a progressive fibrotic disease that is characterized by excessive deposition of extracellular matrix (ECM) components such as collagen and glycoprotein (1). The clinical manifestation and course of SSc are highly heterogeneous. Although the pathogenesis of the disease is complex and remains unknown, vascular damage and inflammation associated with activated innate and adaptive immunity are considered to be involved in the pathological mechanisms underlying the tissue fibrosis, particularly in the skin and visceral organs such as the lung and gastrointestinal tract. Lung fibrosis is a common clinical manifestation of SSc and is the leading cause of death in affected patients.

Injury-induced activation of vascular endothelial cells is the initial and central event of SSc. Activated vascular endothelial cells can induce the perivascular infiltration of inflammatory cells such as CD4^+^ T cells, which can produce high levels of inflammatory cytokines such as interleukin 4 (IL-4) (2, 3). CD4^+^ T cells have recently been shown to be centrally involved in the pathogenesis of SSc. In addition to vasculopathy and fibrosis, activated autoimmunity is another hallmark of SSc. In the preclinical or very early phase, the pathological changes in skin affected by SSc include perivascular edema and inflammatory cell infiltration (4). Increased dermal fibrosis, decreased number of normal capillaries, and loss of skin adnexa are the typical pathological findings that appear in the established stage of SSc (4). In lungs affected by SSc, inflammatory cell infiltration in the interstitium and alveoli precedes evident pulmonary fibrosis (5). These observations strongly suggest that the infiltration of inflammatory cells such as CD4^+^ T cells is the first event in the process leading to the generalized tissue fibrosis in SSc.

Th17 cells are a CD4^+^ T cell subset that secrete primarily IL-17 (IL-17A and IL-17F). Th17 cells and the associated signaling pathways have been shown to be involved in the pathogenesis of many autoimmune and inflammatory diseases, such as rheumatoid arthritis, Sjögren’s syndrome, and type 1 diabetes (6). It has been suggested that the production of IL-17 is increased in SSc patients (7, 8). IL-17 directly promotes the production of IL-6 and IL-8 in human dermal fibroblasts (9). *In vitro* studies have shown that IL-17 increases the proliferation of fibroblasts and IL-1 production in vascular endothelial cells, which suggests a pivotal role of IL-17 in fibrosis and endothelial inflammation (7, 10). Interestingly, many inflammatory cytokines, such as TGF-β, IL-6, and IL-1, which are known to be involved in the pathogenesis of SSc, are mediators that promote Th17 differentiation. This strongly suggests the skewing of CD4^+^ T cell immune response toward Th17 in the disease. However, existing evidences shown in human and experimental animal models of SSc have failed to reach a definite conclusion (11–13). Skin biopsy samples obtained from SSc patients showed the increased population of IL-17+ cells compared to those of healthy controls, and IL-17 attenuated the myofibroblast transdifferentiation, suggesting its antifibrotic role during dermal fibrosis in SSc (11). On the other hand, another study showed the close relationship between SSc disease activity and the number of Th17 cells (14). They also identified that IL-17 derived from Th17 cells promoted collagen production and fibroblast growth (14).

Here, we used two different murine models to examine whether IL-17 activity could augment the fibrotic and inflammatory processes in SSc. *In vivo* studies showed that IL-17 signaling is critically involved in the fibrosis and inflammation processes in a bleomycin (BLM)-induced model of SSc. We also used a chronic graft-versus-host disease (cGVHD) model, another murine model of SSc, to show that blocking IL-17 activity attenuated the clinical severity of cGVHD. IL-1 and IL-17 had synergistic effects on the expression of profibrotic and inflammatory mediators in dermal fibroblasts from humans and mice. We confirmed the antifibrotic and anti-inflammatory potentials of IL-1 receptor antagonist (IL-1Ra) *in vivo*. These findings suggest that an IL-17 cytokine-targeting strategy by blocking IL-1 may be a novel therapeutic strategy to inhibit generalized tissue fibrosis in SSc patients.

## Materials and Methods

### Mice

B6 (H-2kb) and B/c (H-2kd) female mice, 8–10 weeks of age, were purchased from OrientBio (Sungnam, Korea). IL-1Ra knockout (KO) and IL-17 KO mice were obtained from Prof. Yoichiro Iwakura (University of Tokyo, Japan). IL-1Ra-KO mice were backcrossed to IL-17 KO mice over 10 generations, and double KO (DKO) mice were selected for use in polymerase chain reaction (PCR) analysis. The mice were given standard mouse chow (Ralston Purina Co., St. Louis, MO, USA) and water *ad libitum*. All experimental procedures were examined and approved by the Animal Research Ethics Committee of the Catholic University of Korea (Seoul, Korea).

### BLM Treatment

Female mice (aged 6–8 weeks) were anesthetized with isoflurane, and their backs were shaved. Mice were treated daily for 4 weeks with subcutaneous injections of phosphate-buffered saline (PBS) or BLM (Dong-A Pharm, Seoul, Korea). BLM was given at a dose of 1 mg/ml in PBS and was sterilized by filtration before injection into the shaved back skin. The mice (*n* = 5 or 6 per group) were killed by CO_2_ euthanasia on the day after the final treatment, and the back skin and lungs were harvested and fixed in 10% formalin solution for histological analysis.

### Bone Marrow (BM) Transplantation

C57BL/6 recipient mice were intravenously injected with 5 × 10^6^ donor BM cells after lethal irradiation with 800 cGy. Splenocytes (1 × 10^7^ cells) from wild-type (WT) BALB/c or IL-1Ra-KO or IL-1Ra–IL-17-DKO mice were injected intravenously through the tail vein. Mice were monitored for body weight after BM transplantation (BMT) at least twice weekly. Mice were euthanized on day 60 after BMT, and the histopathology of their cGVHD target tissues (skin, lung, liver, and large intestines) was analyzed by investigators who were blinded to the treatment. Organs were harvested, cryoembedded, and sectioned. The sections were fixed in 10% (v/v) buffered formalin and stained with H&E for histological examination.

### Histopathological Assessment

Tissues were fixed in 10% formalin and embedded in paraffin. Sections (6 µm thick) were stained with H&E and Masson’s trichrome (MT). Dermal thickness was examined as previously described (15). The lungs were excised, and the severity of fibrosis was scored as previously described (16). To quantify the amount of collagen in mouse skin and lung tissues, skin and lung was hydrolyzed and used for hydroxyproline assay. Immunohistochemistry was performed using VECTASTAIN ABC kits (Vector Laboratories, Burlingame, CA, USA). Tissues were first incubated with the primary antibodies to IL-1β, IL-6, IL-17, tumor necrosis factor α (TNF-α), transforming growth factor β (TGF-β), and α-smooth muscle actin (α-SMA) overnight at 4°C. The primary antibody was detected with a biotinylated secondary linking antibody, followed by incubation with a streptavidin–peroxidase complex for 1 h. The final color product was developed using DAB chromogen (Dako, Carpinteria, CA, USA). The count of positive cells, identified as a dark-brown deposit in nuclei of lymphocytes, was made in 10 randomly selected high power fields (HPF, 400×; 2.37 mm^2^). To quantify the numbers of infiltrating T cells, B cells, and macrophages, lesional skin sections were stained for CD3, CD22, and F4/80 (Abcam, Cambridge, UK), respectively. The count of positive cells, identified as a discrete membrane staining in cells, was made in 10 randomly selected HPF (400×; 2.37 mm^2^).

### Collagen Assay

To quantify the amount of collagen in mouse skin and lung specimens, hydroxyproline content was measured using a commercial Quickzyme Total Collagen Assay kit (QuickZyme Biosciences). The hydroxyproline assays were performed according to the manufacturer’s protocol.

### Cell Sources and Culture

Mouse skin fibroblasts were isolated by enzymatic digestion of skin from normal BALB/c, as described previously (17). Human dermal fibroblasts were isolated from foreskin tissue obtained from Bucheon St. Mary’s Hospital. The isolation of dermal fibroblasts has been described previously (18). Human experiments were approved by the Institutional Review Board (IRB) of human subjects at Bucheon St. Mary’s Hospital (approval number: HC18TESI0013), The Catholic University of Korea, and conducted accordance with IRB guidelines and regulations. All patients were informed and gave their written consent, and this study was performed in accordance with the Helsinki II Declaration. Dermal fibroblasts obtained from healthy humans and WT BALB/c mice were cultured in Dulbecco’s modified Eagle’s medium (DMEM) supplemented with 10% fetal bovine serum in 150 mm dishes until confluent. Dermal fibroblasts were plated at a density of 5 × 10^4^ cells/well, and the cells were serum starved overnight and then treated with IL-1β (10 ng/ml) or IL-17 (5 ng/ml) for 24 h.

### Fibroblasts and Th17 Lymphocyte Cocultures

Skin fibroblasts from IL-1Ra-KO mice (postnatal days 0–4 mice) were plated at 2 × 10^5^ cells per well in a 12-well culture plates in DMEM medium and allowed to adhere overnight at 37°C in a humidified incubator and 5% CO_2_ atmosphere. The following day, differentiated Th17 cells (2 × 10^6^) were added to skin fibroblasts with or without blocking anti-IL-17 antibody (5 µg/ml). T cells were washed out with fresh media and fibroblasts were then harvested and analyzed for fibrosis-associated gene expression using quantitative real-time PCR.

### Th17 Differentiation of Murine T Cells

Spleens that were isolated from IL-1Ra-KO mice were minced and the red blood cells were lysed with 0.83% ammonium chloride. The cells were filtered through a cell strainer and centrifuged at 1,300 revolutions per minute at 4°C for 5 min. To purify splenic CD4^+^ T cells, the splenocytes were incubated with CD4-coated magnetic beads and isolated using magnetic-activated cell sorting separation columns (Miltenyi Biotec). Positively selected splenic CD4^+^ T cells were stimulated with plate-bound anti-CD3 (0.5 µg/ml), soluble anti-CD28 (1 µg/ml; both from BD Biosciences), anti-interferon-γ (2 µg/ml), anti-IL-4 (2 µg/ml), recombinant transforming growth factor β (2 ng/ml), and recombinant IL-6 (20 ng/ml) (all from R&D Systems) for 3 days to achieve polarization of Th17 cells.

### Real-Time PCR

Messenger RNA (mRNA) was extracted using TRI Reagent (Molecular Research Center, Inc., Cincinnati, OH, USA) according to the manufacturer’s instructions. Complementary DNA was synthesized using a SuperScript Reverse Transcription system (Takara Bio Inc., Kyoto, Japan). A Light-Cycler 2.0 instrument (software version 4.0; Roche Diagnostics, Mannheim, Germany) was used for PCR amplification. All reactions were performed using the LightCycler FastStart DNA Master SYBR Green I mix (Takara), following the manufacturer’s instructions. The following primers were used to amplify mouse genes: for IL-1β, 5′-GGA TGA GGA CAT GAG CAC ATT C-3′ (sense) and 5′-GGA AGA CAG GCT TGT GCT CTG A-3′ (antisense); for IL-6, 5′-ATG CTC CCT GAA TGA TCA CC-3′ (sense) and 5′-TTC TTT GCA AAC AGC ACA GC-3′ (antisense); for IL-17, 5′-CCT-CAA-AGC-TCA-GCG-TGT-CC-3′ (sense) and 5′-GAG-CTC-ACT-TTT-GCG-CCA-AG-3′ (antisense); for TNF-α, 5′-ATG AGC ACA GAA AGC ATG ATC-3′ (sense) and 5′-TAC AGG CTT GTC ACT CGA ATT-3′ (antisense); for MMP-1, 5′-AAC TAC ATT TAG GGG AGA GGT GT-3′ (sense) and 5′-GCA GCG TCA AGT TTA ACT GGA A-3′ (antisense); for MMP-9, 5′-CTG TCC AGA CCA AGG GTA CAG CCT-3′ (sense) and 5′-GAG GTA TAG TGG GAC ACA TAG TGG-3′ (antisense); for Col1A1, 5′-CTC CGG CTC CTG CTC CTC TTA-3′ (sense) and 5′-GCA CAG CAC TCG CCC TCC C-3′ (antisense); and for TGF-β, 5′-GCC TGA GTG GCT GTC TTT TGA-3′ (sense) and 5′-CAC AAG AGC AGT GAG CGC TGA A-3′ (antisense).

The following primers were used to amplify human genes: for IL-6, 5′-AGA CAG CCA CTC ACC TCT TCA G-3′ (sense) and 5′-TTC TGC CAG TGC CTC TTT GCT G-3′ (antisense); for MMP-1, 5′-CTG AAG GTG ATG AAG CAG CC-3′ (sense) and 5′-AGT CCA AGA GAA TGG CCG AG-3′ (antisense); for MMP-9, 5′-CGC AGA CAT CGT CAT CCA GT-3′ (sense) and 5′-GGA TTG GCC TTG GAA GAT GA-3′ (antisense); for Col1A1, 5′-ATG GGA GGA GAG CGT GTG-3′ (sense) and 5′-GAG GTC GGA GAG CAG AGG-3′ (antisense); for TGF-β, 5′-TGC GGC AGC TGT ACA TTG A-3′ (sense) and 5′-TGG TTG TAC AGG GCC AGG A-3′ (antisense). All mRNA levels were normalized to that of β-actin.

### Enzyme-Linked Immunosorbent Assay (ELISA)

The concentrations of IL-1β, IL-1Ra, IL-6, IL-17, and TGF-β in culture supernatants were measured using a sandwich ELISA (DuoSet; R&D Systems, Lille, France). To activate latent TGF-β1 to the immunoreactive form, before being assayed, latent TGF-β1 was activated to the immunoreactive form by acid treatment. Briefly, 125 µl of cell culture supernatant was incubated with 25 µl of 1 N HCl for 10 min at room temperature, neutralized by addition of 25 µl of 1.2 N NaOH/0.5 M 4-(2-hydroxyethyl)-1-piperazineethanesulfonic acid for 10 min. Serum concentrations of IgG antibodies were measured using a commercial ELISA kit (Bethyl Laboratories, Montgomery, TX, USA).

### Statistical Analysis

Data are presented as mean ± SDs of at least three independent experiments or at least three independent samples and for 10 mice in each group. *In vitro* experiments were independently repeated three or more times and each experiment had at least three samples. One-way analysis of variance followed by Bonferroni’s *post hoc* test was used to compare differences between ≥3 groups. The Mann–Whitney *U* test was used to compare numerical data between two groups. To assess the Gaussian distribution and the equality of variance, Shapiro–Wilk test and Levene test were used, respectively. *p* < 0.05 was considered statistically significant. Statistical analysis was performed using IBM SPSS Statistics 20 for Windows (IBM Corp., Armonk, New York, NY, USA).

## Results

### Effects of IL-1β and IL-17 on Gene Expression of IL-6, MMP-9, Col1A1, and TGF-β

**Figure 1:**
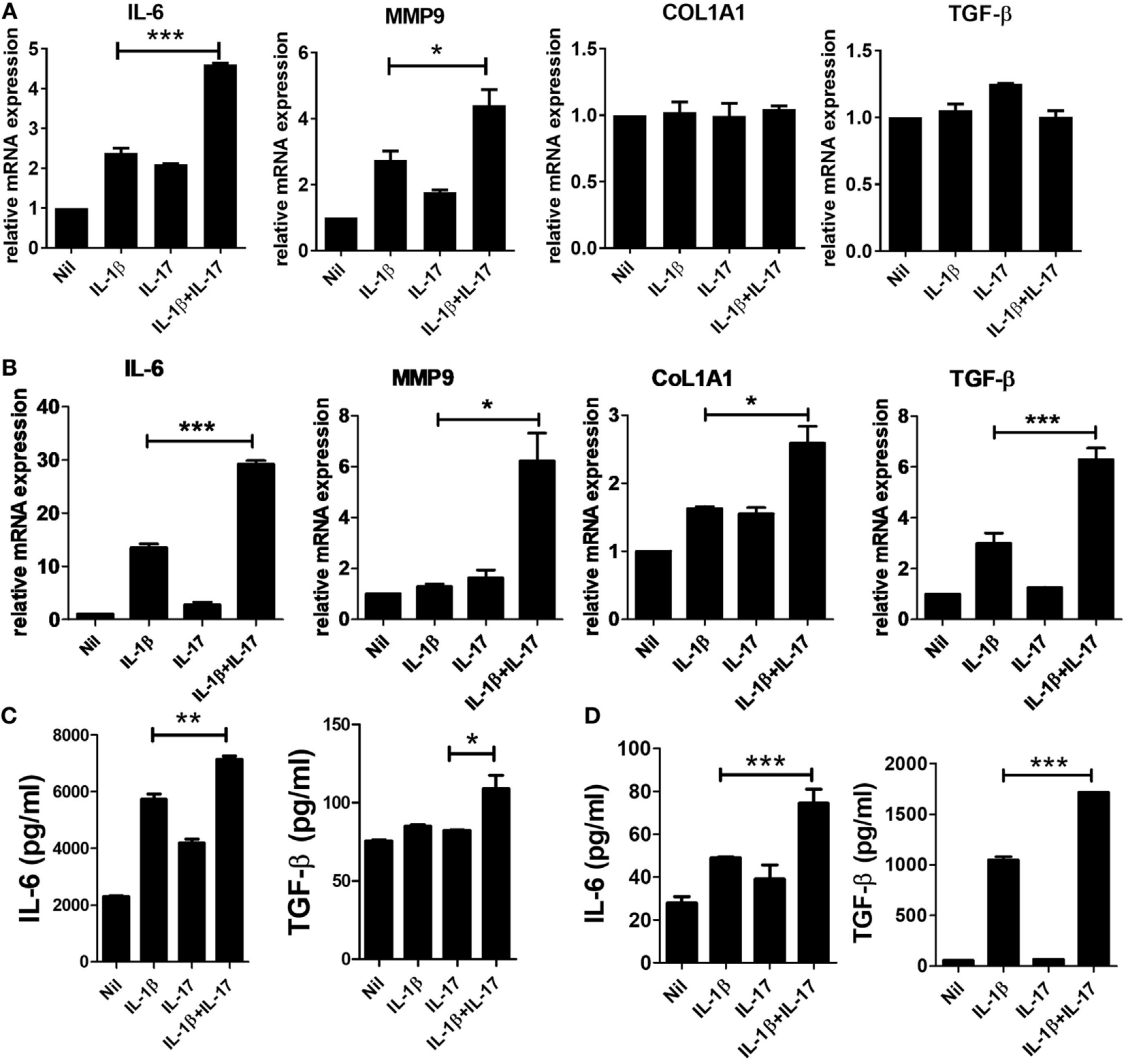
Increased inflammatory and profibrotic cytokine expression induced by IL-1 and IL-17 in dermal fibroblasts. **(A,B)** The expression of messenger RNA for the indicated genes in IL-1- and/or IL-17-treated murine **(A)** and human **(B)** dermal fibroblasts was measured by real-time quantitative polymerase chain reaction analysis. **(C,D)** IL-6 and TGF-β concentrations in culture supernatants of IL-1- and/or IL-17-treated murine **(C)** and human **(D)** dermal fibroblasts were measured by enzyme-linked immunosorbent assay. The data are expressed as the mean ± SEM for three independent experiments per group. *p < 0.05, **p < 0.01, ***p < 0.001.

Many studies have revealed mediators involved in the fibrosis of SSc. IL-6 has been suggested as a treatment target for SSc. IL-6 induces collagen production in skin fibroblasts through JAK2–STAT3-dependent signaling (19, 20). The serum IL-6 level is significantly higher in SSc patients than in healthy people (21), and the level correlates with skin thickness in SSc patients (22). In addition to IL-6, matrix metalloproteinase (MMP), type 1 collagen, and excessive TGF-β activity are revealed to be closely associated with pathological fibrosis in SSc patients (23). MMP-9 is thought to be implicated in fibrotic diseases, such as pulmonary fibrosis (24), and skin fibrosis in SSc patients (25). To study whether IL-1β and IL-17 have a direct effect on IL-6, MMP9, type I collagen, and TGF-β expression, we assessed the expression of profibrotic markers in mouse and human skin fibroblasts. In mouse skin fibroblasts, the mRNA expression levels of IL-6 and MMP-9 were increased by IL-1β or IL-17 treatment. In addition, IL-1β and IL-17 showed synergistic effects on IL-6 and MMP-9 mRNA expression in mouse dermal fibroblasts (Figure 1A). By contrast, the expression levels of *Col1A1* and *TGF*β mRNA were not affected by IL-1β and IL-17.

Next, we examined the effects of IL-1β and IL-17 on the markers mentioned in human dermal fibroblasts. Although each cytokine increased the mRNA expression of IL-6, MMP-9, Col1A1, and TGF-β, the changes were marginal. IL-1β and IL-17 showed significant synergistic effects on IL-6, MMP-9, Col1A1, and TGF-β expression (Figure 1B). To confirm the changes in the protein levels of IL-6 and TGF-β, the concentration of each cytokine was measured in culture supernatants. IL-1β and IL-17 showed synergistic effects on the concentrations of IL-6 and TGF-β in both murine and human dermal fibroblasts, although the effect of each cytokine alone was marginal (Figures 1C,D).

### More Severe Fibrosis of Skin and Lung in IL-1Ra-KO Mice Depends on IL-17 Signaling

**Figure 2:**
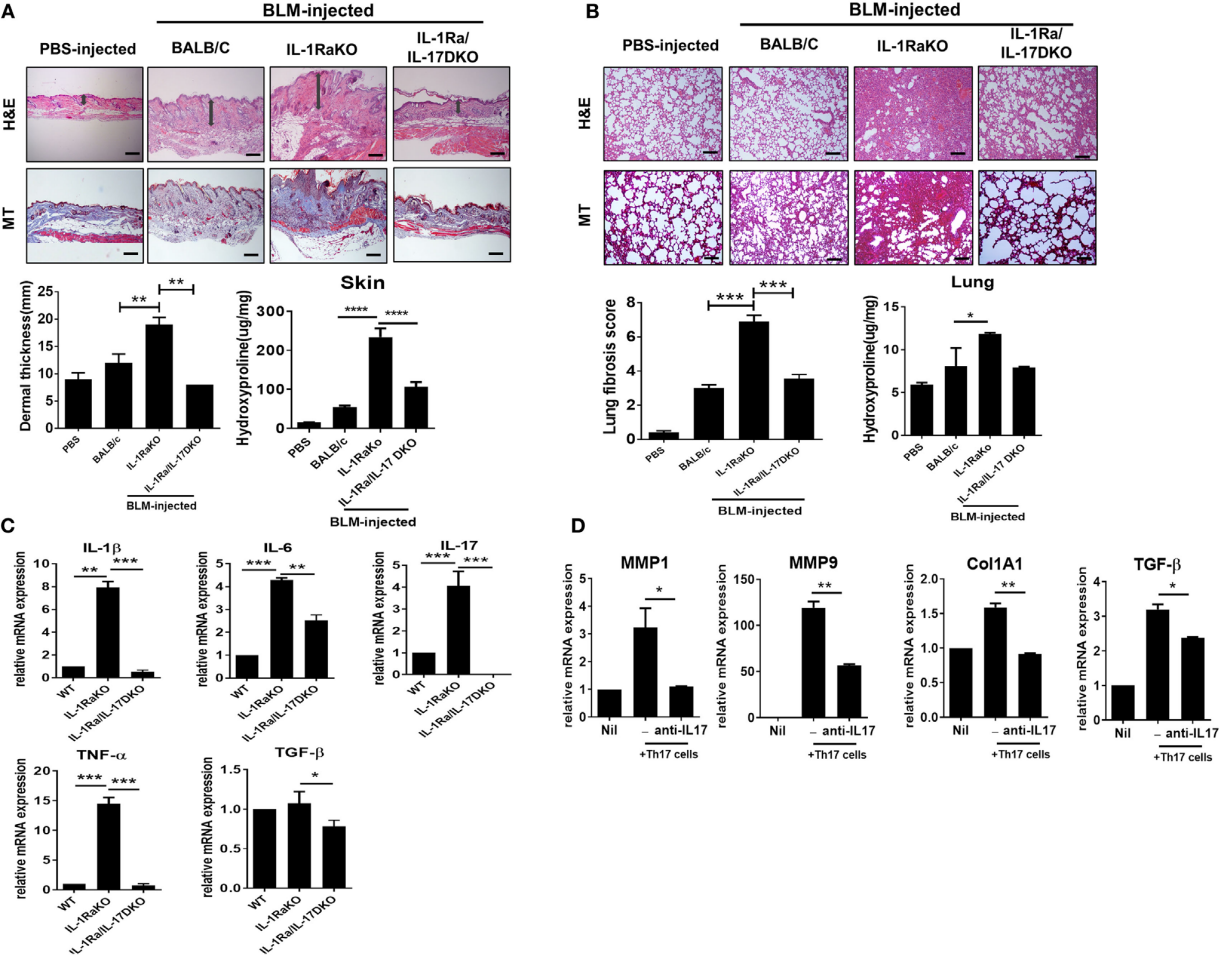
Augmented fibrosis in skin and lung tissue from IL-1 receptor antagonist-deficient (IL-1Ra-KO) mice depends on IL-17 activity. Wild-type (WT) BALB/c mice mice received daily injections of phosphate-buffered saline or bleomycin (BLM) for 28 days (*n* = 6/group). IL-1Ra-KO and IL-1Ra–IL-17-double KO (DKO) mice received daily injections of BLM for 28 days (*n* = 6/group). Then, skin and lung samples were isolated and analyzed. **(A,B)** Left panel, representative sections stained with hematoxylin and eosin, and Masson’s trichrome (MT) in skin **(A)** and lung **(B)**. Original magnification 100×. Scale bar represents 200 µm. Right panel, quantification of dermal thickness in affected skin and pulmonary fibrosis, showing greater dermal thickness and lung fibrosis in IL-1Ra-KO mice injected with BLM relative to those in WT mice and a significant reduction in IL-1Ra–IL-17-DKO mice relative to those in IL-1Ra-KO mice. Hydroxyproline content in skin and lung tissues from each group of mice. Values are the mean ± SEM of six mice per group. **(C)** mRNA levels of IL-1β, IL-6, IL-17, TNF-α, and TGF-β in skin samples from WT, IL-1Ra-KO, and IL-1Ra–IL-17-DKO mice treated with BLM as determined by real-time quantitative polymerase chain reaction analysis. **(D)** mRNA levels of MMP1, MMP9, Col1A1, and TGFβ in dermal fibroblasts (2 × 10^5^ cells) of IL-1Ra-KO mice that were cocultured with *in vitro* differentiated Th17 cells (2 × 10^6^ cells) in the absence or presence of anti-IL-17 antibody (5 µg/ml). The data are expressed as the mean ± SEM for three independent experiments per group. *p < 0.05, **p < 0.01, ***p < 0.001.

IL-1Ra is an endogenous inhibitor of IL-1 activity that competes with IL-1 (IL-1α and IL-1β) for binding to the IL-1 receptor. IL-1Ra-KO mice on a BALB/c background were developed by Iwakura et al. for use as an animal model of spontaneous arthritis (26). Iwakura and colleagues identified that IL-17 production is markedly induced in IL-1Ra-KO mice and that the inflammatory arthritis in that animal model requires IL-17 and T cells (27). To investigate the pathophysiological role of IL-17 during the development of dermal and pulmonary fibrosis, the subcutaneous BLM-induced SSc model was induced in WT BALB/c, IL-1Ra-KO mice, and DKO mice (IL-1Ra–IL-17-DKO). After 4 weeks of BLM injection, skin and lung tissues were extracted and analyzed. Histological analyses of skin and lung samples stained with H&E or MT (for collagen identification) showed that BLM injection in WT mice increased dermal thickness, caused greater ECM deposition in the dermis, and induced greater fibrosis of adipose tissue in the subcutaneous layer relative to the results following injection of PBS (Figure 2A). And, BLM-induced skin and lung fibrosis was significantly greater in IL-1Ra-KO mice than in WT BALB/c mice (Figures 2A,B). Intriguingly, IL-1Ra–IL-17-DKO mice showed significantly attenuated fibrosis and less collagen deposition in skin and lung tissues than did IL-1Ra-KO mice (Figures 2A,B). Deposition of collagen in skin and lung tissues, quantified by measuring the level of hydroxyproline, also significantly increased in IL-1Ra-KO mice compared to WT BALB/c mice (Figures 2A,B). This increase in hydroxyproline content shown in IL-1Ra-KO mice was significantly reduced in IL-1Ra–IL-17-DKO mice (Figures 2A,B). Next, to examine the changes in inflammatory cytokines, quantitative real-time PCR was used to analyze skin samples from each group of mice. mRNA expression of IL-1β, IL-6, IL-17, and TNF-α was much higher in IL-1Ra-KO mice than in WT mice (Figure 2C). And, the mRNA levels of all of these genes were significantly lower in IL-1Ra–IL-17-DKO mice than in IL-1Ra-KO mice (Figure 2C). By contrast, TGF-β expression in affected skin did not differ between the WT mice and IL-1Ra-KO mice, although there was marginal difference between IL-1Ra-KO mice and IL-1Ra–IL-17-DKO mice, which suggests that TGF-β signaling is not associated with IL-1β- and IL-17-induced augmented fibrosis (Figure 2C).

Furthermore, to identify the effect of IL-17 on the altered fibrosis severities shown in IL-1Ra-KO mice and IL-1Ra–IL-17-DKO mice, dermal fibroblasts isolated from IL-1Ra-KO mice were cocultured with *in vitro* differentiated Th17 cells that were generated using splenic CD4^+^ T cells from IL-1Ra-KO mice. After 3 days of coculture, T cells were washed out and dermal fibroblasts were then harvested and analyzed for fibrosis-associated gene expressions. The results showed that *MMP1, MMP9, Col1A1*, and *TGF*β mRNA expressions were significantly increased in the dermal fibroblasts cultured with Th17 cells compared to those in single cultured system (Figure 2D). And, this increase was attenuated or abolished by anti-IL-17 antibody with IL-17 blocking property (Figure 2D). Taken together, these findings suggest that the increased dermal thickness and collagen deposition shown in BLM-treated IL-1Ra-KO mice is largely dependent on the activity of IL-17 located in the downstream signal of IL-1β.

### The Expression of Inflammatory and Fibrotic Cytokines in Affected Skin

**Figure 3:**
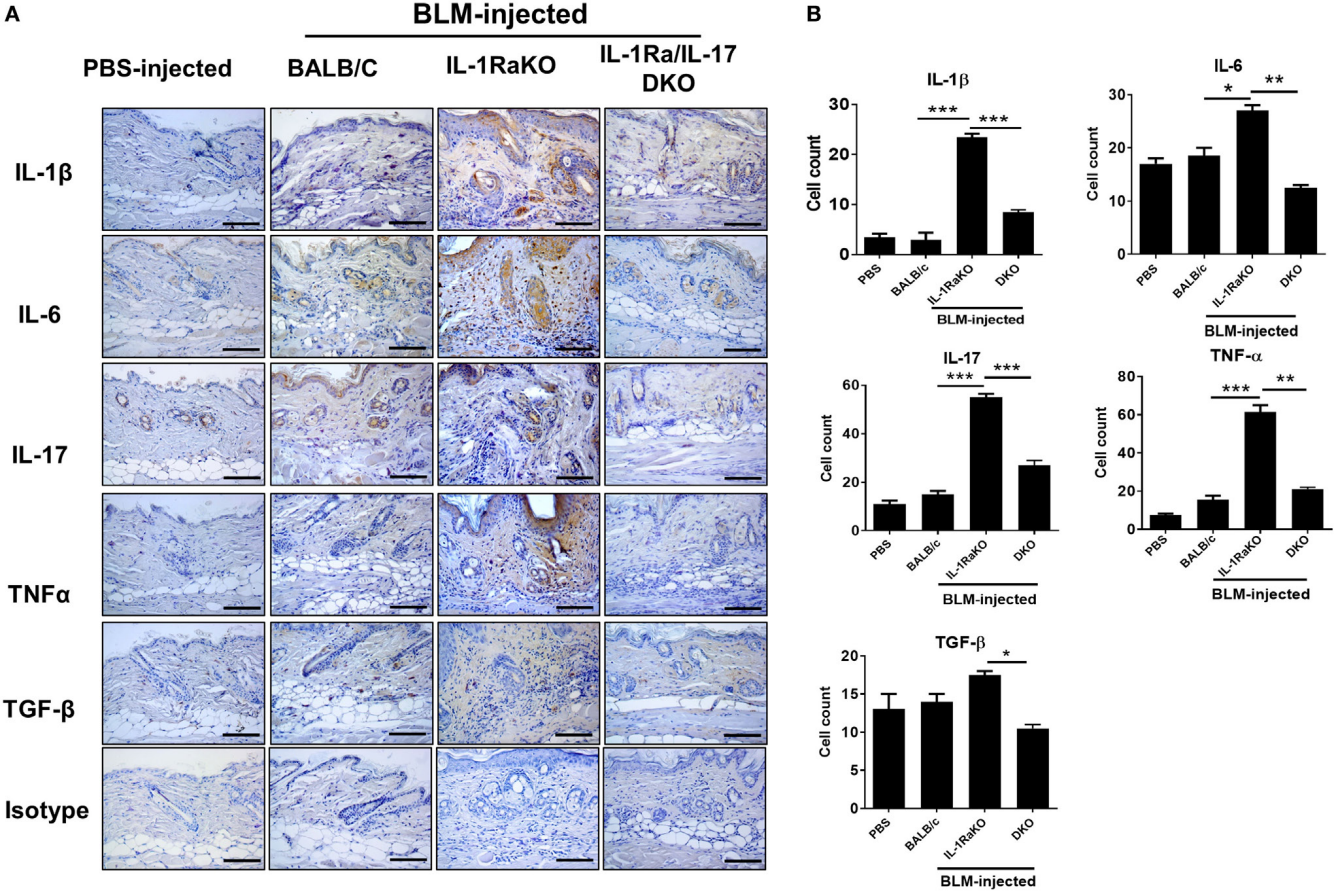
Inflammatory mediators in lesional skin. **(A)** Representative skin tissue section from the phosphate-buffered saline- or Bleomycin-injected mice were stained for IL-1β, IL-6, IL-17, TNF-α, and TGF-β. Original magnification 400×. Scale bar represents 100 µm. **(B)** Positive cells are indicated in the bar graphs. The data are expressed as the mean ± SEM for three independent experiments per group. *p < 0.05, **p < 0.01, ***p < 0.001.

Immunohistochemical staining was performed on skin samples from the four animal groups (PBS-injected negative control, BLM-induced fibrosis in WT BALB/c mice, BLM-induced fibrosis in IL-1Ra-KO mice, and BLM-induced fibrosis in IL-1Ra–IL-17-DKO mice). The numbers of IL-1β-, IL-6-, IL-17-, and TNF-α-expressing cells were significantly greater in BLM-injected IL-1Ra-KO mice than in WT BALB/c mice. By contrast, the number of TGF-β-expressing cells did not differ between these two groups (Figures 3A,B). These results suggest that augmented tissue fibrosis and inflammation caused by excessive IL-1β activity are associated with IL-6, IL-17, and TNF-α, but not with TGF-β. Further, to study whether the attenuated fibrosis in IL-1Ra–IL-17-DKO mice was associated with inflammatory and fibrotic mediators, immunohistochemical staining results of IL-1Ra/IL-17 DKO mice were compared with those of IL-1Ra-KO. The expression levels of IL-1β, IL-6, IL-17, TNF-α, and TGF-β were all significantly lower in IL-1Ra–IL-17-DKO mice than in IL-1Ra-KO mice (Figures 3A,B).

### Inhibition of Inflammatory and Fibrotic Molecules and Suppression of the Infiltration of T and B Cells and Macrophages by Blocking IL-17 Activity in the Mouse Model of SSc

**Figure 4:**
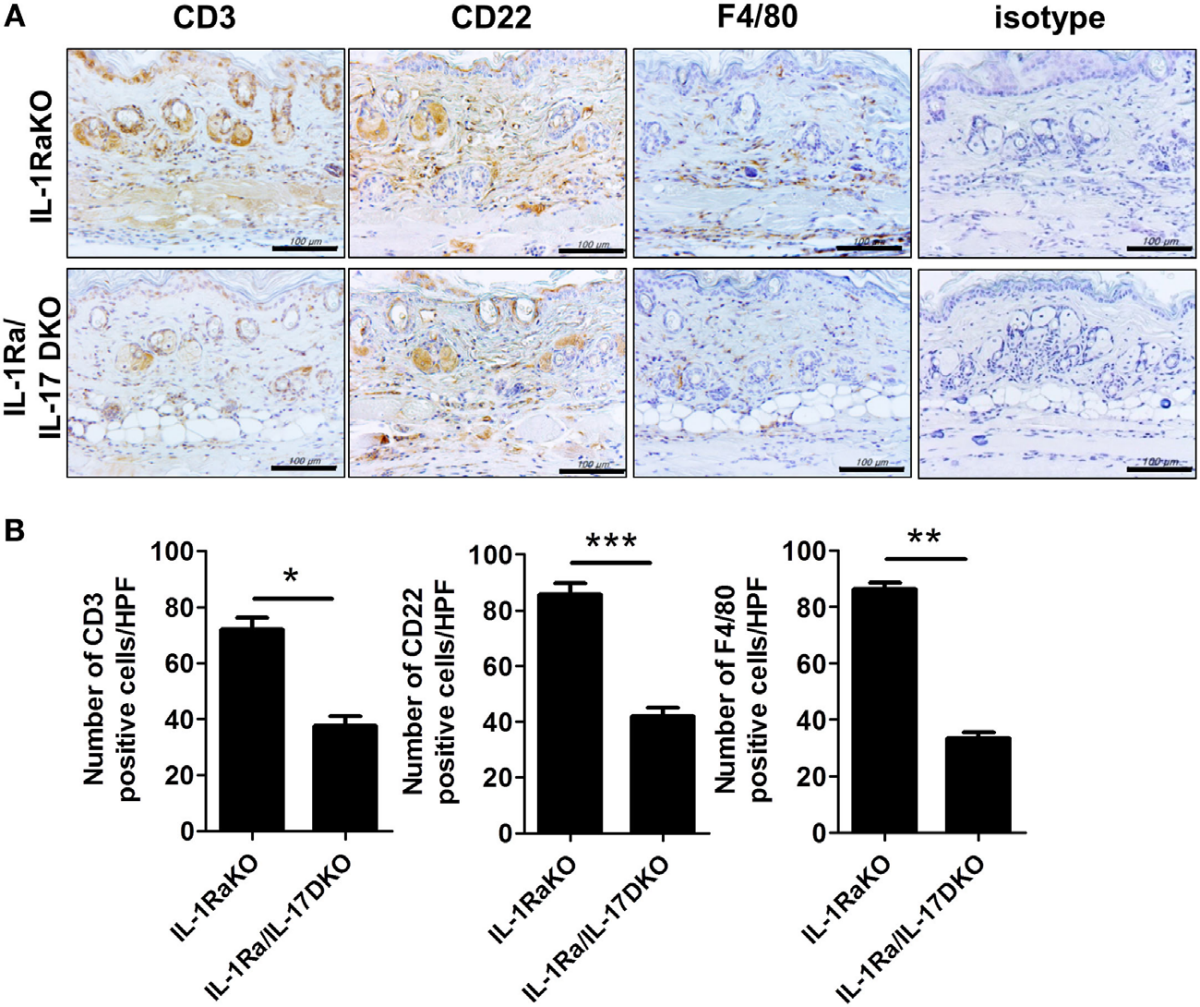
Inflammatory cell infiltration into lesional skin. **(A)** Representative sections of lesional skin stained for CD3, CD22, and F4/80 by immunohistochemistry. Scale bar represents 100 µm. **(B)** The number of infiltrating T cells (CD3^+^), B cells (CD22^+^), and macrophages (F4/80^+^) per HPF are shown in bar graphs as the mean ± SEM for three independent experiments per group. *p < 0.05, **p < 0.01, ***p < 0.001.

To identify the altered populations of immune cells, the populations of T cells, B cells, and macrophages infiltrating into dermal tissues in each group of mice were evaluated by immunohistochemical staining against CD3 (T cells), CD22 (B cells), and F4/80 (macrophages). Fewer cells of each type were detected in IL-1Ra–IL-17-DKO mice than in IL-1Ra-KO mice (Figures 4A,B).

### Antifibrotic and Anti-Inflammatory Properties of IL-1Ra in BLM-Induced SSc Model

**Figure 5:**
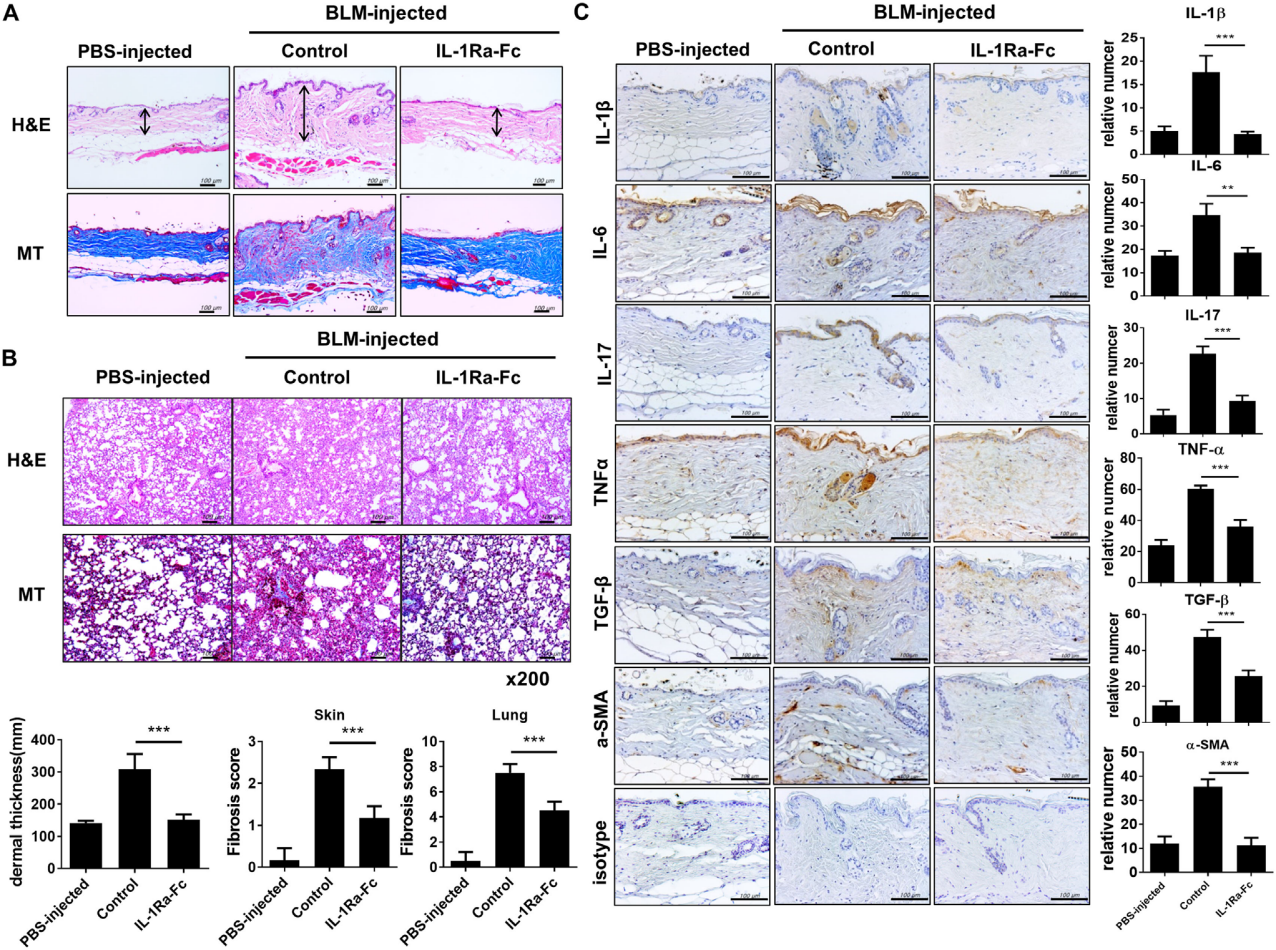
Antifibrotic effects of IL-1 receptor antagonist in the bleomycin (BLM)-induced fibrosis model. **(A,B)** C57BL/6 mice received a daily injection of phosphate-buffered saline or BLM for 28 days (*n* = 5/group). The mice with BLM-injected fibrosis were divided into a group that received the IL-1 receptor antagonist (anakinra) and a group that did not receive anakinra (*n* = 5/group). Anakinra (100 mg/kg) was injected intraperitoneally three times per week for 2 weeks (from day 15 to day 28) after the start of BLM injection. Representative sections stained with hematoxylin and eosin and Masson’s trichrome (MT) in skin **(A)** and lung **(B)** show the reduced dermal thickness [arrows in **(A)**] and attenuated skin and lung fibrosis following anakinra. Scale bar represents 100 µm (skin) and original magnification 200× (lung). Quantification of dermal thickness in affected skin and fibrosis score of skin and lung are shown in bar graphs as the mean ± SEM for five independent experiments per group. ***p < 0.001. **(C)** Immunohistochemical staining for the frequency of IL-1β-, IL-6-, IL-17-, TNF-α-, TGF-β-, and α-SMA-positive cells in lesional skin. Left panel; positive immunoreactivity appears as a brown color. Scale bar represents 100 µm. Right panel; the data are expressed as the mean ± SEM for three independent experiments per group. Scale bar represents 100 µm. **p < 0.01, ***p < 0.001.

We investigated the therapeutic effect of an IL-1Ra (anakinra) in the BLM-induced fibrosis model in mice with a C57BL/6 background. After 4 weeks of BLM injection, skin and lung tissues were extracted and analyzed. Both BLM-injected C57BL/c mice showed evident fibrosis of the skin and lungs (Figure 5A). To ascertain whether anakinra could attenuate the fibrosis in the skin and lung in BLM-induced fibrosis mice, 100 mg/kg anakinra was injected intraperitoneally three times per week for 2 weeks (from day 15 to day 28) after the start of BLM administration. Anakinra treatment in this BLM-induced fibrosis model caused marked reduction in collagen deposition and fibrosis of skin and lung tissues (Figures 5A,B). Next, the expression of inflammatory cytokines was examined in the skin tissues of each group. Activated dermal myofibroblasts are key effector cells in SSc that synthesize the excessive production of collagen, which causes fibrosis in affected organs, especially in the skin and lung (28, 29). The expression levels of IL-1β, IL-6, IL-17, TNF-α, TGF-β, and α-SMA (as a myofibroblast marker) were all decreased by anakinra treatment, which suggested anti-inflammatory and antifibrotic effects of the IL-1Ra in this BLM-induced fibrosis model (Figure 5C).

### Antifibrotic and Anti-Inflammatory Properties of IL-1Ra in Sclerotic Chronic GVHD Model

**Figure 6:**
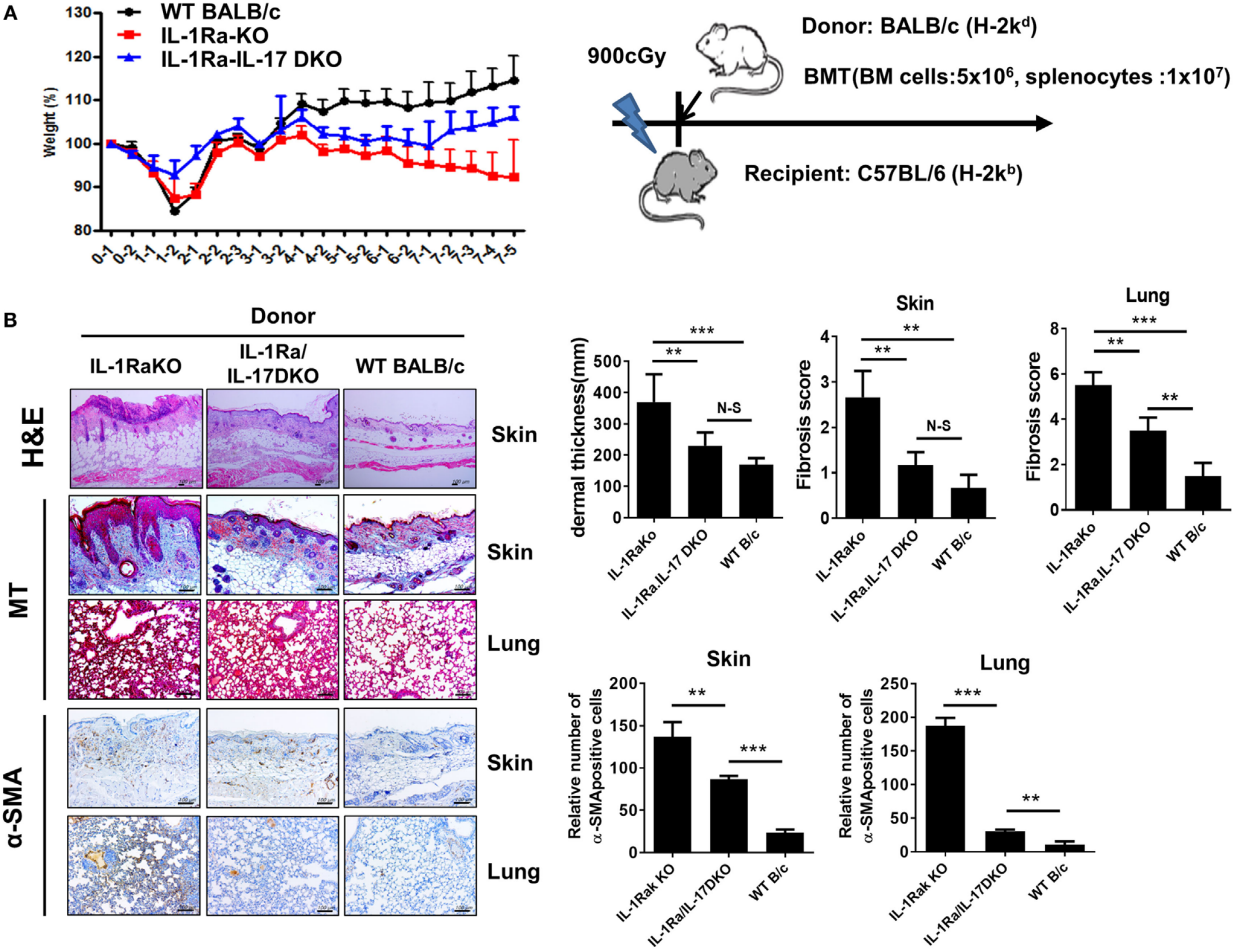
Antifibrotic effects of IL-17 blocking in sclerodermatous chronic graft-versus-host disease (cGVHD) models. **(A)** Weight was monitored in mice with cGVHD. Schematic representation of the murine sclerodermatous cGVHD model. Irradiated recipient C57BL/6 mice received 5 × 10^6^ bone marrow (BM) cells and splenocytes (1 × 10^7^ cells) from one of three different kinds of donor mice with a BALB/c background: wild-type (WT) BALB/c, IL-1 receptor antagonist-deficient (IL-1Ra-KO), and IL-1 receptor antagonist and IL-17 double-knockout (IL-1Ra–IL-17-double KO). **(B)** Left panel, representative sections stained with hematoxylin and eosin, and Masson’s trichrome (MT) and immunohistochemical staining for the frequency of α-SMA-positive cells in skin and lung tissues after BM transplantation (BMT; *n* = 10/group). Positive immunoreactivity appears as a brown color. The data are representative of two independent experiments. Tissues isolated at week 8 after BMT. Scale bar represents 100 µm. Right panel, dermal thickness, skin and lung fibrosis score, and the frequency of α-SMA-positive cells in skin and lung tissues are expressed as the mean ± SEM from two independent experiments (*n* = 10 mice per group). **p < 0.01, ***p < 0.001.

Systemic sclerosis is an immune disorder characterized by fibrosis and autoimmunity. However, the BLM-induced fibrosis model reflects only the fibrosis and inflammation of SSc and cannot fully represent the role of autoimmunity, which is a major treatment target of SSc. cGVHD provides another model to study SSc and produces progressive fibrosis in the skin, liver, gastrointestinal tract, and lung. The cGVHD model reflects all of the major pathologies of SSc, including inflammation, fibrosis, and autoimmunity. We used the cGVHD model to confirm the pathological role of IL-1β–IL-17 signaling in the autoimmunological aspects of SSc. Irradiated recipient C57BL/6 mice received donor BM cells and splenocytes (1 × 10^7^ cells) from one of three different kinds of donor mice with a BALB/c background: WT BALB/c, IL-1Ra-KO, or IL-1Ra–IL-17-DKO mice (Figure 6A). Weight loss was greater in recipient mice that received BM cells and splenocytes from IL-1Ra-KO mice but was less in mice that received cells from IL-1Ra–IL-17-DKO mice (Figure 6A). Skin and lung are major target organs in cGVHD. These organs were isolated from the three groups of cGVHD mice at 60 days after BMT and then analyzed histologically. Similar to the patterns of weight changes, more severe inflammation was observed in the skin, and lung of recipient mice that received BM cells and splenocytes from IL-1Ra-KO mice than in those that received BM and cells from WT mice (Figure 6B). The accelerated tissue inflammation and fibrosis were reversed by blocking IL-17 (in IL-1Ra–IL-17-DKO mice). Taken together, these findings in two different animal models suggest that excessive IL-1β activity exacerbates target tissue fibrosis and inflammation by augmenting downstream IL-17 activity.

## Discussion

In the present study, we investigated the pathophysiological roles of IL-1 and its downstream cytokine IL-17 in fibrosis and inflammation of target tissues in SSc. We found that IL-1 and IL-17 activity was increased in skin and lung tissues of murine models of SSc. We present evidence that IL-1-mediated skin and lung fibrosis depends on IL-17 activity and is associated with IL-6 and TNF-α activity, but not with TGF-β. Our *in vitro* study showed that IL-1β and IL-17 have synergistic stimulatory effects on IL-6 and MMP-9 production in murine and human skin fibroblasts. This pathophysiological role of the IL-1–IL-17 axis was confirmed in two different murine models of SSc in our present study.

Here, we found that IL-1 activity may contribute to fibrosis by inducing IL-6 and TGF-β expression in skin fibroblast and resulted in increased fibrosis in BLM-induced SSc model. We identified that exacerbated fibrosis and inflammation by IL-1 activity shown *in vivo* is largely dependent on IL-17. IL-1β, one of proinflammatory cytokine, has been unrevealed to promote collagen synthesis and aggregate fibrosis, suggesting its pro-fibrotic property (30, 31). Previous studies have demonstrated the abnormally high activities of IL-1 as well as IL-1 receptor in SSc patients (32, 33), indicating the pathophysiological roles of IL-1 signal transduction in SSc. Recently, Wilson et al. reported that intratracheal administration of IL-1β induces pulmonary fibrosis similar to BLM-induced fibrosis model, which is dependent on IL-17 activity (34). Their study is consistent with our findings showing that BLM-induced tissue fibrosis was exacerbated by systemically increased IL-1 activity through IL-1Ra depletion. Here, we demonstrated that IL-1 and IL-17, located in the downstream signal of IL-1, are important therapeutic targets for fibrosis inhibition of SSc. Furthermore, we identified that blocking the IL-1 receptor *in vivo* by anakinra administration significantly attenuated BLM-induced tissue fibrosis.

In our results, mRNA expression of type 1 collagen was increased when dermal fibroblasts isolated from IL-1Ra-KO mice were cocultured with murine Th17 cells (as shown in Figure 2D). Despite of recent recognition of the pathophysiological roles of IL-17 or Th17 cells in SSc, there have been conflicting results observed between the mice and human data. As with our findings, murine skin fibroblasts increased collagen synthesis by IL-17 treatment in a dose-dependent manner (15). Also *in vivo*, hypodermal thickness was reduced by IL-17 deficiency in a murine model of SSc (15). Most of the mice results have shown the pro-fibrotic effects of IL-17 or Th17 in SSc, both *in vitro* and *in vivo* experiments (34–37). On the other hand, in human, existing evidences have demonstrated the conflicting conclusion. In patients with SSc, the number of IL-17-expressing cells and mRNA expressions of IL-17 are increased in SSc skin than in healthy control skin (7, 11). However, treatment with IL-17 or Th17 cell supernatants in human skin fibroblast did not increase the production of type 1 collagen and connective tissue growth factor (7, 38, 39), whereas IL-17 enhanced fibroblast proliferation (7). Thus, further studies are needed to clarify the effect of Th17 or IL-17 on human fibroblasts, regarding the progressive fibrosis of SSc target organs, such as skin and lung.

Few treatment options can effectively suppress or reverse progressive tissue fibrosis in SSc patients. These options include methotrexate, cyclophosphamide, or autologous hematopoietic stem cell transplantation (40–42). However, the therapeutic efficacy of these strategies is limited because of the restricted duration of the time when the condition can be improved, the marginal effectiveness of these treatments, and the very narrow selection of candidate patients (41–43). In addition, the potential risks seem to outweigh the effectiveness of these treatments. Significant side effects include: liver toxicity, pancytopenia, teratogenicity, and lung toxicity, which are associated with methotrexate or cyclophosphamide treatment; and procedure-related mortality, associated with the hematopoietic stem cell transplantation (44). Despite the emerging concept of the use of molecular targets, such as endothelin-1, for the treatment of fibrosis in SSc, the clinical efficacy and safety profile of antifibrotic treatments including tyrosine kinase inhibitors such as imatinib await confirmation (45–48).

In our present study, we identified the pathophysiological role of IL-1 and its downstream IL-17 activity in SSc-associated inflammation and fibrosis using two different murine models of the disease. We found that increased IL-1 activity aggravated fibrosis in the skin and lung tissues of SSc mice and that fibrosis exacerbated by this increased IL-1 activity could be reversed by blocking IL-17. Our findings implicate IL-1β and its downstream IL-17 signaling in the tissue fibrosis, inflammation, and autoimmunity involved in SSc. Our results imply that IL-1–IL-17 signaling may be a novel therapeutic target for attenuating or reversing ongoing inflammation and fibrosis in SSc patients.

## Ethics Statement

Human dermal fibroblasts were isolated from foreskin tissue obtained from Bucheon St. Mary’s Hospital. The isolation of dermal fibroblasts has been described previously (18). Human experiments were approved by the Institutional Review Board (IRB) of human subjects at Bucheon St. Mary’s Hospital (approval number: HC18TESI0013), The Catholic University of Korea, and conducted accordance with IRB guidelines and regulations. All patients were informed and gave their written consent and this study was performed in accordance with the Helsinki II Declaration.

## Author Contributions

All the authors were involved in drafting the article or revising it critically for important intellectual content, and all authors approved the final version to be published. M-LC and S-HP had full access to all of the data in the study and takes responsibility for the integrity of the data and the accuracy of the data analysis. M-JP, S-JM, S-HP, and M-LC contributed to study conception, study design, data acquisition, analysis, and interpretation and drafted the manuscript. E-JL, K-AJ, E-KK, and D-SK contributed to data acquisition, analysis, and interpretation. J-HL, S-KK, and J-KM contributed to analysis and interpretation of data.

## Conflict of Interest Statement

The authors declare that the research was conducted in the absence of any commercial or financial relationships that could be construed as a potential conflict of interest.

## References

[1] Gabrielli A Avvedimento EV Krieg T Scleroderma. N Engl J Med (2009) 360:1989–2003. 10.1056/NEJMra0806188 19420368

[2] Guiducci S Distler O Distler JH Matucci-Cerinic M Mechanisms of vascular damage in SSc – implications for vascular treatment strategies. Rheumatology (Oxford) (2008) 47(Suppl 5):v18–20. 10.1093/rheumatology/ken267 18784130

[3] Parel Y Aurrand-Lions M Scheja A Dayer JM Roosnek E Chizzolini C. Presence of CD4+CD8+ double-positive T cells with very high interleukin-4 production potential in lesional skin of patients with systemic sclerosis. Arthritis Rheum (2007) 56:3459–67. 10.1002/art.22927 17907151

[4] Prescott RJ Freemont AJ Jones CJ Hoyland J Fielding P. Sequential dermal microvascular and perivascular changes in the development of scleroderma. J Pathol (1992) 166:255–63. 10.1002/path.1711660307 1517881

[5] Harrison NK Myers AR Corrin B Soosay G Dewar A Black CM Structural features of interstitial lung disease in systemic sclerosis. Am Rev Respir Dis (1991) 144:706–13. 10.1164/ajrccm/144.3_Pt_1.706 1892314

[6] Singh RP Hasan S Sharma S Nagra S Yamaguchi DT Wong DT Th17 cells in inflammation and autoimmunity. Autoimmun Rev (2014) 13:1174–81. 10.1016/j.autrev.2014.08.019 25151974

[7] Kurasawa K Hirose K Sano H Endo H Shinkai H Nawata Y Increased interleukin-17 production in patients with systemic sclerosis. Arthritis Rheum (2000) 43:2455–63. 10.1002/1529-0131(200011)43:11<2455:aid-anr12>3.0.co;2-k 11083268

[8] Rodriguez-Reyna TS Furuzawa-Carballeda J Cabiedes J Fajardo-Hermosillo LD Martinez-Reyes C Diaz-Zamudio M Th17 peripheral cells are increased in diffuse cutaneous systemic sclerosis compared with limited illness: a cross-sectional study. Rheumatol Int (2012) 32:2653–60. 10.1007/s00296-011-2056-y 21789610

[9] Fossiez F Djossou O Chomarat P Flores-Romo L Ait-Yahia S Maat C T cell interleukin-17 induces stromal cells to produce proinflammatory and hematopoietic cytokines. J Exp Med (1996) 183:2593–603. 10.1084/jem.183.6.2593 8676080 PMC2192621

[10] Xing X Yang J Yang X Wei Y Zhu L Gao D IL-17A induces endothelial inflammation in systemic sclerosis via the ERK signaling pathway. PLoS One (2013) 8:e85032. 10.1371/journal.pone.0085032 24376862 PMC3871633

[11] Truchetet ME Brembilla NC Montanari E Lonati P Raschi E Zeni S Interleukin-17A+ cell counts are increased in systemic sclerosis skin and their number is inversely correlated with the extent of skin involvement. Arthritis Rheum (2013) 65:1347–56. 10.1002/art.37860 23335253

[12] Lei L Zhao C Qin F He ZY Wang X Zhong XN. Th17 cells and IL-17 promote the skin and lung inflammation and fibrosis process in a bleomycin-induced murine model of systemic sclerosis. Clin Exp Rheumatol (2016) 34(Suppl 100):14–22. 26750756

[13] Chizzolini C Dufour AM Brembilla NC. Is there a role for IL-17 in the pathogenesis of systemic sclerosis? Immunol Lett (2018) 195:61–7. 10.1016/j.imlet.2017.09.007 28919455

[14] Yang X Yang J Xing X Wan L Li M. Increased frequency of Th17 cells in systemic sclerosis is related to disease activity and collagen overproduction. Arthritis Res Ther (2014) 16:R4. 10.1186/ar4430 24398084 PMC3979142

[15] Okamoto Y Hasegawa M Matsushita T Hamaguchi Y Huu DL Iwakura Y Potential roles of interleukin-17A in the development of skin fibrosis in mice. Arthritis Rheum (2012) 64:3726–35. 10.1002/art.34643 22833167

[16] Yoshizaki A Iwata Y Komura K Ogawa F Hara T Muroi E CD19 regulates skin and lung fibrosis via toll-like receptor signaling in a model of bleomycin-induced scleroderma. Am J Pathol (2008) 172:1650–63. 10.2353/ajpath.2008.071049 18467694 PMC2408424

[17] Rogers KM Black DH Eberle R. Primary mouse dermal fibroblast cell cultures as an in vitro model system for the differential pathogenicity of cross-species herpesvirus papio 2 infections. Arch Virol (2007) 152:543–52. 10.1007/s00705-006-0865-1 17122896

[18] Pajoum Shariati SR Shokrgozar MA Vossoughi M Eslamifar A. In Vitro co-culture of human skin keratinocytes and fibroblasts on a biocompatible and biodegradable scaffold. Iran Biomed J (2009) 13:169–77. 19688023

[19] Duncan MR Berman B. Stimulation of collagen and glycosaminoglycan production in cultured human adult dermal fibroblasts by recombinant human interleukin 6. J Invest Dermatol (1991) 97:686–92. 10.1111/1523-1747.ep12483971 1940439

[20] Khan K Xu S Nihtyanova S Derrett-Smith E Abraham D Denton CP Clinical and pathological significance of interleukin 6 overexpression in systemic sclerosis. Ann Rheum Dis (2012) 71:1235–42. 10.1136/annrheumdis-2011-200955 22586157

[21] Hasegawa M Sato S Fujimoto M Ihn H Kikuchi K Takehara K. Serum levels of interleukin 6 (IL-6), oncostatin M, soluble IL-6 receptor, and soluble gp130 in patients with systemic sclerosis. J Rheumatol (1998) 25:308–13. 9489824

[22] Sato S Hasegawa M Takehara K. Serum levels of interleukin-6 and interleukin-10 correlate with total skin thickness score in patients with systemic sclerosis. J Dermatol Sci (2001) 27:140–6. 10.1016/S0923-1811(01)00128-1 11532378

[23] Sonnylal S Denton CP Zheng B Keene DR He R Adams HP Postnatal induction of transforming growth factor beta signaling in fibroblasts of mice recapitulates clinical, histologic, and biochemical features of scleroderma. Arthritis Rheum (2007) 56:334–44. 10.1002/art.22328 17195237

[24] Betsuyaku T Fukuda Y Parks WC Shipley JM Senior RM. Gelatinase B is required for alveolar bronchiolization after intratracheal bleomycin. Am J Pathol (2000) 157:525–35. 10.1016/s0002-9440(10)64563-4 10934155 PMC1850142

[25] Kim WU Min SY Cho ML Hong KH Shin YJ Park SH Elevated matrix metalloproteinase-9 in patients with systemic sclerosis. Arthritis Res Ther (2005) 7:R71–9. 10.1186/ar1454 15642145 PMC1064883

[26] Horai R Saijo S Tanioka H Nakae S Sudo K Okahara A Development of chronic inflammatory arthropathy resembling rheumatoid arthritis in interleukin 1 receptor antagonist-deficient mice. J Exp Med (2000) 191:313–20. 10.1084/jem.191.2.313 10637275 PMC2195765

[27] Nakae S Saijo S Horai R Sudo K Mori S Iwakura Y. IL-17 production from activated T cells is required for the spontaneous development of destructive arthritis in mice deficient in IL-1 receptor antagonist. Proc Natl Acad Sci U S A (2003) 100:5986–90. 10.1073/pnas.1035999100 12721360 PMC156313

[28] Hinz B The extracellular matrix and transforming growth factor-beta1: tale of a strained relationship. Matrix Biol (2015) 47:54–65. 10.1016/j.matbio.2015.05.006 25960420

[29] Gerber EE Gallo EM Fontana SC Davis EC Wigley FM Huso DL Integrin-modulating therapy prevents fibrosis and autoimmunity in mouse models of scleroderma. Nature (2013) 503:126–30. 10.1038/nature12614 24107997 PMC3992987

[30] Kolb M Margetts PJ Anthony DC Pitossi F Gauldie J. Transient expression of IL-1beta induces acute lung injury and chronic repair leading to pulmonary fibrosis. J Clin Invest (2001) 107:1529–36. 10.1172/jci12568 11413160 PMC200196

[31] Thomay AA Daley JM Sabo E Worth PJ Shelton LJ Harty MW Disruption of interleukin-1 signaling improves the quality of wound healing. Am J Pathol (2009) 174:2129–36. 10.2353/ajpath.2009.080765 19389930 PMC2684178

[32] Umehara H Kumagai S Murakami M Suginoshita T Tanaka K Hashida S Enhanced production of interleukin-1 and tumor necrosis factor alpha by cultured peripheral blood monocytes from patients with scleroderma. Arthritis Rheum (1990) 33:893–7. 10.1002/art.1780330619 2363741

[33] Kawaguchi Y Harigai M Hara M Suzuki K Kawakami M Ishizuka T Increased interleukin 1 receptor, type I, at messenger RNA and protein level in skin fibroblasts from patients with systemic sclerosis. Biochem Biophys Res Commun (1992) 184:1504–10. 10.1016/S0006-291X(05)80053-1 1375465

[34] Wilson MS Madala SK Ramalingam TR Gochuico BR Rosas IO Cheever AW Bleomycin and IL-1beta-mediated pulmonary fibrosis is IL-17A dependent. J Exp Med (2010) 207:535–52. 10.1084/jem.20092121 20176803 PMC2839145

[35] Mi S Li Z Yang HZ Liu H Wang JP Ma YG Blocking IL-17A promotes the resolution of pulmonary inflammation and fibrosis via TGF-beta1-dependent and -independent mechanisms. J Immunol (2011) 187:3003–14. 10.4049/jimmunol.1004081 21841134

[36] Gasse P Riteau N Vacher R Michel ML Fautrel A di Padova F IL-1 and IL-23 mediate early IL-17A production in pulmonary inflammation leading to late fibrosis. PLoS One (2011) 6:e23185. 10.1371/journal.pone.0023185 21858022 PMC3156735

[37] Simonian PL Roark CL Wehrmann F Lanham AK Diaz del Valle F Born WK Th17-polarized immune response in a murine model of hypersensitivity pneumonitis and lung fibrosis. J Immunol (2009) 182:657–65. 10.4049/jimmunol.182.1.657 19109199 PMC2766086

[38] Nakashima T Jinnin M Yamane K Honda N Kajihara I Makino T Impaired IL-17 signaling pathway contributes to the increased collagen expression in scleroderma fibroblasts. J Immunol (2012) 188:3573–83. 10.4049/jimmunol.1100591 22403442

[39] Brembilla NC Montanari E Truchetet ME Raschi E Meroni P Chizzolini C. Th17 cells favor inflammatory responses while inhibiting type I collagen deposition by dermal fibroblasts: differential effects in healthy and systemic sclerosis fibroblasts. Arthritis Res Ther (2013) 15:R151. 10.1186/ar4334 24289089 PMC3979123

[40] Kowal-Bielecka O Fransen J Avouac J Becker M Kulak A Allanore Y Update of EULAR recommendations for the treatment of systemic sclerosis. Ann Rheum Dis (2017) 76:1327–39. 10.1136/annrheumdis-2016-209909 27941129

[41] Tashkin DP Elashoff R Clements PJ Goldin J Roth MD Furst DE Cyclophosphamide versus placebo in scleroderma lung disease. N Engl J Med (2006) 354:2655–66. 10.1056/NEJMoa055120 16790698

[42] van Laar JM Farge D Sont JK Naraghi K Marjanovic Z Larghero J Autologous hematopoietic stem cell transplantation vs intravenous pulse cyclophosphamide in diffuse cutaneous systemic sclerosis: a randomized clinical trial. JAMA (2014) 311:2490–8. 10.1001/jama.2014.6368 25058083

[43] Pope JE Bellamy N Seibold JR Baron M Ellman M Carette S A randomized, controlled trial of methotrexate versus placebo in early diffuse scleroderma. Arthritis Rheum (2001) 44:1351–8. 10.1002/1529-0131(200106)44:6<1351:aid-art227>3.0.co;2-i 11407694

[44] Lateef O Shakoor N Balk RA. Methotrexate pulmonary toxicity. Expert Opin Drug Saf (2005) 4:723–30. 10.1517/14740338.4.4.723 16011450

[45] Denton CP Merkel PA Furst DE Khanna D Emery P Hsu VM Recombinant human anti-transforming growth factor beta1 antibody therapy in systemic sclerosis: a multicenter, randomized, placebo-controlled phase I/II trial of CAT-192. Arthritis Rheum (2007) 56:323–33. 10.1002/art.22289 17195236

[46] Pope J McBain D Petrlich L Watson S Vanderhoek L de Leon F Imatinib in active diffuse cutaneous systemic sclerosis: results of a six-month, randomized, double-blind, placebo-controlled, proof-of-concept pilot study at a single center. Arthritis Rheum (2011) 63:3547–51. 10.1002/art.30549 21769850

[47] Bournia VK Evangelou K Sfikakis PP. Therapeutic inhibition of tyrosine kinases in systemic sclerosis: a review of published experience on the first 108 patients treated with imatinib. Semin Arthritis Rheum (2013) 42:377–90. 10.1016/j.semarthrit.2012.06.001 22789835

[48] Khanna D Denton CP Merkel PA Krieg T Le Brun FO Marr A Effect of macitentan on the development of new ischemic digital ulcers in patients with systemic sclerosis: DUAL-1 and DUAL-2 randomized clinical trials. JAMA (2016) 315:1975–88. 10.1001/jama.2016.5258 27163986

